# RipleyGUI: software for analyzing spatial patterns in 3D cell distributions

**DOI:** 10.3389/fninf.2013.00005

**Published:** 2013-04-09

**Authors:** Kristin Hansson, Mehrdad Jafari-Mamaghani, Patrik Krieger

**Affiliations:** ^1^Department of Neuroscience, Karolinska InstitutetStockholm, Sweden; ^2^Mathematical Statistics, Centre for Mathematical Sciences, Lund UniversityLund, Sweden; ^3^Department of Biosciences and Nutrition, Karolinska InstitutetHuddinge, Sweden; ^4^Department of Mathematics, Stockholm UniversityStockholm, Sweden

**Keywords:** Ripley's K-function, spatial point pattern, software, cell distribution, neuroanatomical method

## Abstract

The true revolution in the age of digital neuroanatomy is the ability to extensively quantify anatomical structures and thus investigate structure-function relationships in great detail. To facilitate the quantification of neuronal cell patterns we have developed *RipleyGUI*, a MATLAB-based software that can be used to detect patterns in the 3D distribution of cells. *RipleyGUI* uses Ripley's *K*-function to analyze spatial distributions. In addition the software contains statistical tools to determine quantitative statistical differences, and tools for spatial transformations that are useful for analyzing non-stationary point patterns. The software has a graphical user interface making it easy to use without programming experience, and an extensive user manual explaining the basic concepts underlying the different statistical tools used to analyze spatial point patterns. The described analysis tool can be used for determining the spatial organization of neurons that is important for a detailed study of structure-function relationships. For example, neocortex that can be subdivided into six layers based on cell density and cell types can also be analyzed in terms of organizational principles distinguishing the layers.

## Introduction

Determining the spatial distribution of cells is important for projects aiming at large scale re-constructions of neuronal networks (Heintz, 2004; Markram, 2006; Smith, 2007; Helmstaedter et al., 2008; Lichtman et al., 2008; Defelipe, 2010; Oberlaender et al., 2012).

If a certain neurological disorder can be correlated with a change in the cell distribution, this data is of course not sufficient to explain the disease, but can rather help understand how connectivity might have been affected (Landau et al., 2004; Landau and Everall, 2008; Armstrong, 2010). Analyzing changes in connectivity can be much more painstaking than simply analyzing a re-distribution in soma locations. The changes that these alterations in soma distributions cause for the connectivity can subsequently be analyzed using computational modeling of large-scale anatomical networks (Eberhard et al., 2006; Gleeson et al., 2007; Koene et al., 2009; Zubler and Douglas, 2009; Lang et al., 2011). On a larger scale it is known that the brain can be divided into different anatomical and functional areas, but less is known about the functional significance of ordered structures on a smaller scale such as for example the dendrite bundles from layer 5B cells (Krieger et al., 2007) or even cortical columns (Horton and Adams, 2005; Rockland, 2010). To fully explore the potential of the large data sets, which can be obtained using imaging and digitization techniques, it is necessary to develop automatized analysis tools (Wearne et al., 2005; Bjaalie, 2008; Oberlaender et al., 2009; Meijering, 2010; Meyer et al., 2010). In this paper we describe such a software tool and exemplify its use for analyzing neuron distributions in neocortex.

A spatial point pattern is a set of locations, or events, within a specified region (Diggle, 2003). The events are irregularly placed and are modeled as the result of an unknown underlying stochastic process, referred to as a spatial point process. We can think of the distribution of neurons as the result of one such process. Analysis of spatial point patterns is a mathematical tool that allows us to obtain a quantified readout of the organization of neurons.

When exploring the properties of an unknown spatial distribution, the first step is to look at the intensity. The intensity, *lambda*, can be estimated as the average number of events per unit volume. A spatial distribution is also characterized by its second-order properties, that is, how events distribute in relation to each other. Ripley's *K*-function is a method for exploring second-order properties in *n*-dimensions (Ripley, 1979, 1988; Baddeley et al., 1993; Diggle, 2003; Mattfeldt, 2005; Eglen et al., 2008; Jafari-Mamaghani et al., 2010; Millet et al., 2011). The three-dimensional case requires more elaborated methods for edge correction (Baddeley et al., 1993; Eglen et al., 2008; Jafari-Mamaghani et al., 2010). We provide a MATLAB-based software for various analytical uses of Ripley's *K*-function using the 3D edge correction term developed in (Jafari-Mamaghani et al., 2010) which in contrast to other edge correction terms (Baddeley et al., 1993) is based on the exact evaluation of volumes rather than calculations of surface areas.

Examples of software for spatial analysis of 2D and 3D data, respectively, is PAST and SpPACK, which has an impressive number of functions (Hammer et al., 2001; Perry, 2004), and SA3D and PASSaGE (Eglen et al., 2008; Rosenberg and Anderson, 2011) that evaluates Voronoi tessellations, nearest neighbor distance and estimates Ripley's *K*-function. The software presented in this paper, *RipleyGUI*, focuses on using Ripley's *K*-function and in contrast to existing software includes statistical tools that allow the user to easily compare cell distributions, thus providing methods for a more thorough analysis of the data. Furthermore *RipleyGUI* handles sets of data for analyzing the mean and variance of the estimated *K*-function within a data set, and through comparison with distributions following complete spatial randomness (CSR), the statistical significance level of all findings can be calculated. An important complement and improvement to existing software are thus the statistical tools implemented in *RipleyGUI* to determine statistically significant differences. *RipleyGUI* is written in MATLAB which is commonly used by experimental scientists and can thus easily be integrated with other analysis plugins.

## Implementation

### Computing environment

*RipleyGUI* has been developed using MATLAB 7.1. The only requirement to run *RipleyGUI* is to have MATLAB, preferably version 7.0 or later, with the Statistics toolbox. *RipleyGUI* has been tested on Windows XP, Windows Vista, Ubuntu, and Mac OS X. Nevertheless, given the cross-platform nature of MATLAB, it can be used with any Unix, Macintosh, or Windows environment. The software is distributed as an open-source software with a user manual.

*RipeyGUI* requires only basic experience and knowledge of MATLAB. The user should be familiar with the MATLAB environment and MATLAB path definitions. *RipleyGUI* is started by typing “RipleyGUI” in the MATLAB command window (a detailed explanation is given in the accompanying manual). The user can now interact with a graphical interface without the need of any implementation of MATLAB commands. Further analysis can be done by embedding the generated data into MATLAB's workspace.

### Data input/output

The state of *RipleyGUI* including all calculated functions can be saved in native MATLAB format at any time to be retrieved later. All figures can be opened in separate MATLAB windows from where they can be saved in all formats supported by MATLAB, such as .jpg, .png, or .fig. *RipleyGUI* loads neuron distributions from single files or from folders with files. When single files are loaded the defaults file format is “^*^.ascii” but selecting in the import dialog “All files” also “.txt” and “.csv” files can be imported. If files are imported in the import “Set” option the imported files must be in the “^*^.ascii” format. This enables the user to keep comments in “.txt” format in the same folder as the files that will be analyzed with RipleyGUI. Necessary in both cases is that the file has no headings and three columns (corresponding to the x, y, and z values) separated by comma, tab, or space.

### Reference distributions

To help the user get familiar with the *K*-function and how it behaves for different types of distributions *RipleyGUI* contains functions for generating some basic distributions with a user defined volume and intensity. The distributions are based on the intensity parameter and the underlying stochastic process.

The reference distributions include (1) the homogenous Poisson process, (2) the simple Poisson inhibition process, (3) the Poisson cluster process, and (4) the Poisson inhibited cluster process. These processes are also elaborated on in the *RipleyGUI* User Manual.

#### The homogenous poisson process (CSR)

In the Homogenous Poisson Process, events are placed randomly and independently in a 3D region. The distribution of the events is assumed to follow CSR. They can be generated for different values of lambda, the intensity of the process. The total number of events depends on lambda (λ) and the size of the volume (V), (number of events = λ× V).

#### Simple poisson inhibition process

In an inhibited or sparse distribution events are less likely to appear close to other events. A simple inhibition distribution is created through generation of independent events where any event closer than a certain distance to an earlier event is discarded. New events are generated until the desired intensity is reached. This type of distribution can be used to take the cell size into account when mimicking a situation where cells are placed randomly and independently, and where events cannot be closer than the diameter of the cells. The constraint on event proximity limits the maximum number of events (see *RipleyGUI* Manual).

#### Poisson cluster process

In a clustered, or aggregated, point pattern distribution most events are closer to their neighbors than expected comparing to a distribution under CSR. A Poisson cluster distribution is created from randomly distributed parent events, which independently from each other create offspring events. Seeding locations of the offspring are independently and identically distributed according to an exponential family distribution. Only the offspring are part of the final distribution (Diggle, 2003). Offspring with a position outside the volume are placed on the other side of the volume, that is, the distribution is wrapped along its diagonal.

#### Poisson inhibited cluster process

This distribution combines the properties of the inhibited and clustered Poisson processes. This can be a way to take the cell size into account when mimicking a situation where neurons are clustered.

### Stationarity

#### Station

As an optional feature in *RipleyGUI*, the Station routine rotates a sample distribution using a rotation matrix, minimizing the volume needed to contain the events in the distribution. The rotation is performed in 2D, the thinnest dimension is ignored during rotation. This is suitable for distribution regions where parts of the region are vacant.

#### Divide

Another *RipleyGUI* routine Divide cuts a distribution in pieces along its longest side. (If the longest and second-longest sides are equal, Divide will have no effect.) This will help station to create stationary subsets and obtain a more uniformly shaped sample domain.

### Data analysis

#### Ripley's K-function

*RipleyGUI* estimates the *K*-function in three dimensions with edge correction, and displays plots of how the sample domain distribution deviates from its expected value. One strength of the program is that it also manages sets of distributions and allows the user to estimate the average *K*-function of the set and compare it to the expected values of *K*-functions for a set of distributions following CSR. The average is weighted so that distributions with more events influence the average proportionally.

#### Bootstrapping confidence intervals

When working with sets of distributions, *RipleyGUI* uses a bootstrapping method to create confidence intervals around the estimated *K*-function average. The upper and lower intervals within which 95% of the $\hat{K}$-functions can be expected to fall are displayed.

#### Comparing with CSR

To quantify the deviation of a distribution from CSR, *RipleyGUI* creates a comparison set of distributions. The comparison set has the same size and intensity as the distribution being tested but consists of distributions following CSR. The sample distribution is compared to the distributions following CSR and *RipleyGUI* will test whether or not the hypothesis that the sample distribution follows CSR can be rejected for different values of distance *t*. In calculations with a relatively low number of events the simulated CSR distribution can appear more inhibited than actually expected. This occurs as a consequence of how the boundaries are defined. Boundaries are defined as the maximum span between events in each dimension and that might be smaller than the region in which the cell data was acquired, especially in distributions that have few events. This, however, affects the sample distribution and the simulated distributions following CSR both in the same direction.

#### Comparing between data sets

To facilitate for the user to make comparisons between sample sets *RipleyGUI* displays the estimated *K*-functions for up to three data sets in the same plot. By visually inspecting the overlap between the estimated *K*-functions the user will get an overview of for which *t*-values the $\hat{K}$-functions differ. To confirm the difference between sets *RipleyGUI* is able to perform between-group comparisons (Figures 4, 5).

The between-group comparison is based on the hypothesis that two sets are based on identical point pattern distributions. Under this hypothesis, replacing a distribution in a set with a distribution from the other set should not affect the weighted average $\hat{K}$-functions. To verify this hypothesis, sets with the same number of samples as the original data set, chosen randomly from both sets are created using replacement. This procedure is done 5000 times. A score using a function of sum of squares, is calculated for each of the 5000 re-samplings and the real sets (Diggle, 2003). The verification of the hypothesis is then reduced to investigating whether or not the score based on the real sets is likely to have been produced by the scores under the hypothesis (Diggle, 2003; Jafari-Mamaghani et al., 2010).

### Intended use and future directions of the software

This paper accompanies the first release of *RipleyGUI* showing how it can be used to analyze the 3D distribution of cells. Examples from neuroanatomy where this type of analysis can be used includes the analysis of the spatial distribution of neocortical layer 5B cell clusters (White and Peters, 1993; Krieger et al., 2007) and interneurons (Yanez et al., 2005), the vertical alignment of neurons in frontal cortex (Semendeferi et al., 2011), and the distribution of cells in the retina (Novelli et al., 2007). The software is released with an extensive user manual. Future developments of the program includes (1) re-programming in C to increase the speed of the edge correction, and (2) add the possibility to use Ripley's *K*-function in 3D for a cross-correlation analysis of two different populations, thus investigating if cells from two different populations are attracted or repelled from each other.

## Application

We used *RipleyGUI* to analyze spatial properties of genetically labeled layer 5 pyramidal neurons in neocortex. This section can be used as a guide to interpret the results from *RipleyGUI*.

### Run RipleyGUI

**Figure 1:**
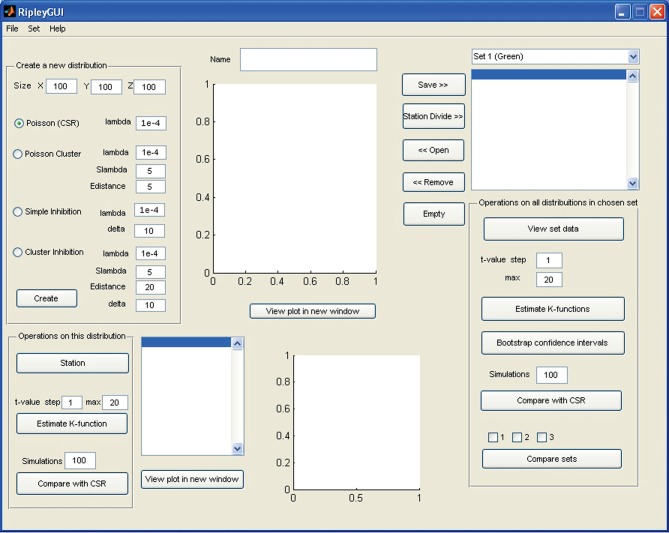
**Screenshot of the opening screen of RipleyGUI.** The upper left panel (Create a new distribution) is allocated for distribution simulations. Each of the four reference distributions (see section Reference distributions) can be tuned with intensity and other parameters. The simulated distributions are displayed in the upper center panel (Name). The lower left panel (Operations on this distribution) is designed for analysis of the distribution on display in the upper central panel (Name). The right panel (Operations on all distributions in a chosen set) is designed for saving, managing, and analyzing single or multiple data sets. The results of all the analysis can be viewed inside or outside of *RipleyGUI* depending on the user's preference. All analysis-related parameters are tunable in their corresponding panels.

**Figure 2:**
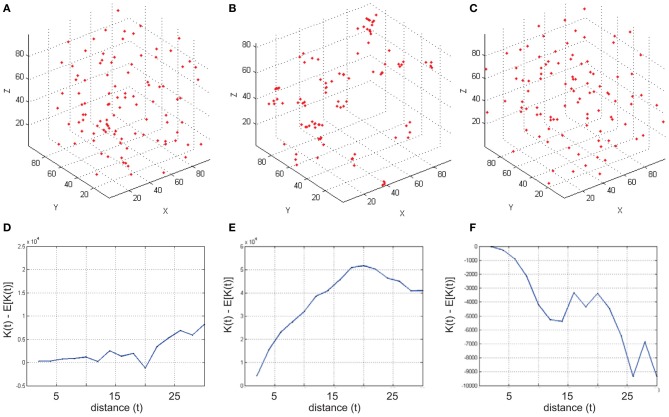
**Examples of simulated reference cell distributions. (A,D)** The Homogenous Poisson Process [Complete spatial randomness (CSR)]. The difference $\hat{K}$(*t*) – *E*[$\hat{K}$(*t*)] is close to 0 and we cannot discard that the sample distribution is following CSR. **(B,E)** $\hat{K}$(*t*) – *E*[$\hat{K}$(*t*)] is positive indicating aggregation. (**C,F**) $\hat{K}$(*t*) – *E*[$\hat{K}$(*t*)] is negative indicating inhibition (dispersion). Data was generated using a *t*-value step 2, and max 30.

To run *RipleyGUI*, type RipleyGUI in your MATLAB command window; this will open the window shown in Figure 1. It is now possible to load test distributions as explained in the User Manual. As an introduction to spatial point patterns the user can first use the reference distributions (section Reference distributions) to study the *K*-function. Spatial point patterns can be divided into three main categories of patterns (Diggle, 2003): aggregation, where events tend to attract other events (clustering); inhibition, where events tend to repel other events and hence create a more regular pattern (dispersion); and CSR where events are distributed randomly. A plot of these three different built-in distributions and the *K*-function analysis of these distributions are shown in Figure 2. In *RipleyGUI* the estimated *K*-function is often displayed as the difference between the estimated K-function [$\hat{K}$(*t*)] and the expected (*E*[$\hat{K}$(*t*)]) K-function to make deviations from the CSR pattern more noticeable. When the estimated *K*-function value is similar to the expected value from a distribution following CSR (*E*[$\hat{K}$(t)]) the difference |($\hat{K}$(*t*) – *E*[$\hat{K}$(*t*)])| is close to 0 and we cannot discard that the sample distribution is following CSR (Figures 2A,D); when the difference is positive (the values of the estimated *K*-function are higher than the expected value from a distribution following CSR) it indicates aggregation (Figures 2B,E); when the difference is negative it indicates inhibition (Figures 2C,F). An estimation of $\hat{K}$(t) (or in general any stochastic quantity) is based on sample observations under given assumptions that might not always be fulfilled. The expectation (*E*[$\hat{K}$(t)]) of a stochastic quantity is the mean value of the quantity under fulfilled assumptions over the entire population.

### Using RipleyGUI on experimental data

#### Corticostriatal cells in visual and somatosensory barrel cortex

**Figure 3:**
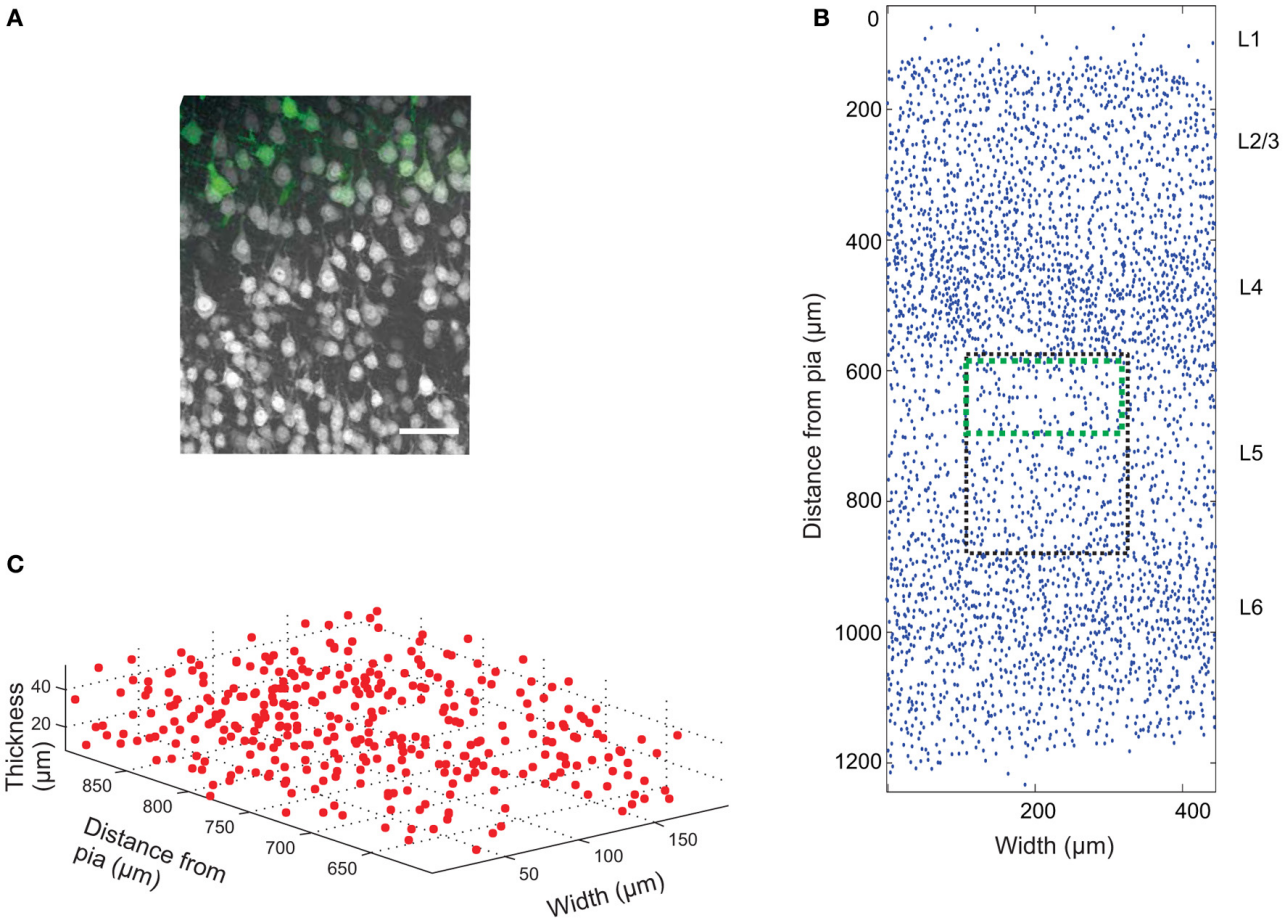
**Cell count data. (A)** Confocal image (z-projection) of a brain slice showing neurons (gray) labeled with Neuronal Nuclei (NeuN) antibodies, and genetically EGFP-labeled layer 5a corticostriatal pyramidal cells (green). Scale bar 50 μm. **(B)** 2D projection of manually placed markers indicating the position of NeuN-labeled cell bodies in a brain slice of the somatosensory mouse cortex cut in the coronal plane. Pia matter is at *y* = 0, and the y-axis is distance from pia matter; x-axis is the width of the tissue slice. The six cortical layers are labeled L1 (Layer 1), etc. The black box shows the approximate position of the image in **(A)** and the green box the approximate position of the EGFP-labeled cells. A sub-section of the image is plotted in 3D in **(C)**.

**Figure 4:**
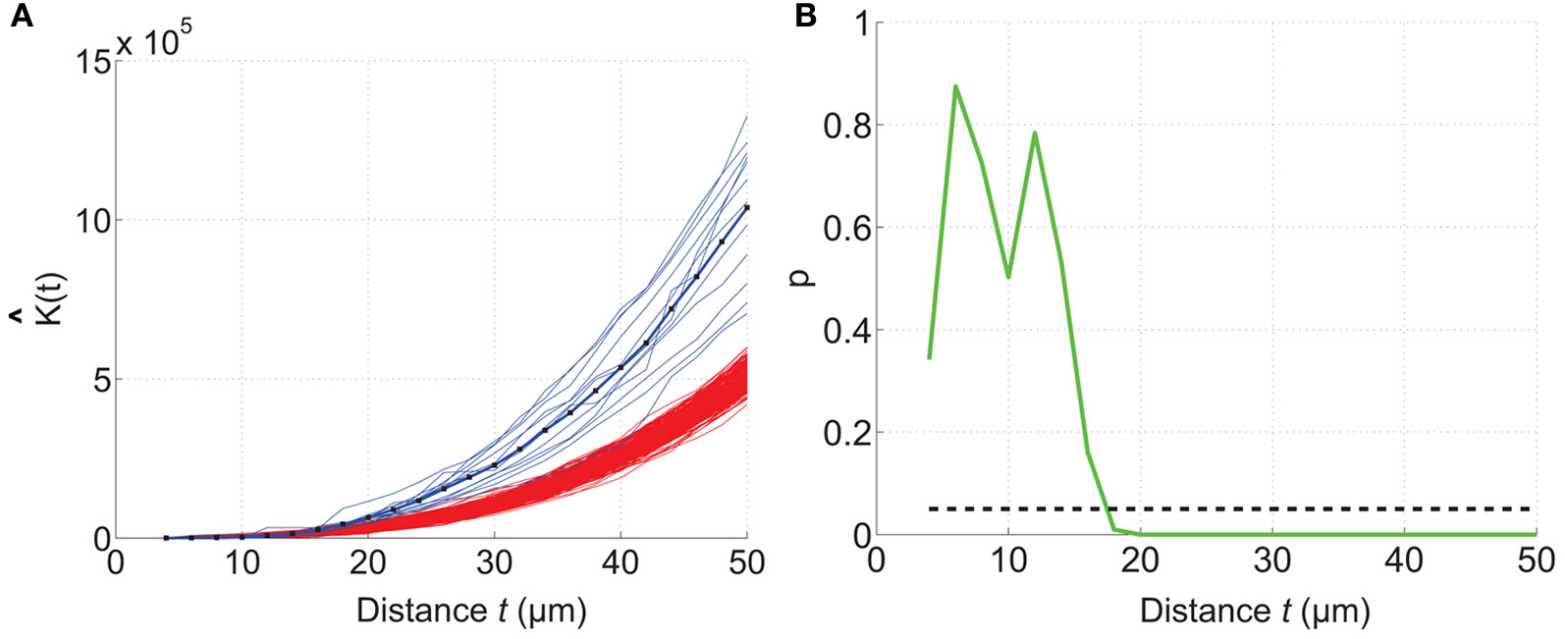
**Comparing a test distribution with a CSR distribution.** Etv-pyramid distributions in visual cortex (vc) are not randomly distributed. The samples (*n* = 6) have been divided and rotated (using Divide and Station) to obtain stationarity. 200 CSR distributions were generated, and used to create a confidence interval for the CSR hypothesis. **(A)** The estimated *K*-function for etv-pyramids (blue lines) compared to simulated CSR distributions (red lines). The *K*-function is estimated for *t*-values between 4 and 50 μm with a 2 μm step size. **(B)** *P*-values from the hypothesis test of CSR. For *t* = 18 μm the etv-pyramid distributions differs from CSR with 95% significance. These types of graphs can be generated with the *RipleyGUI*.

**Figure 5:**
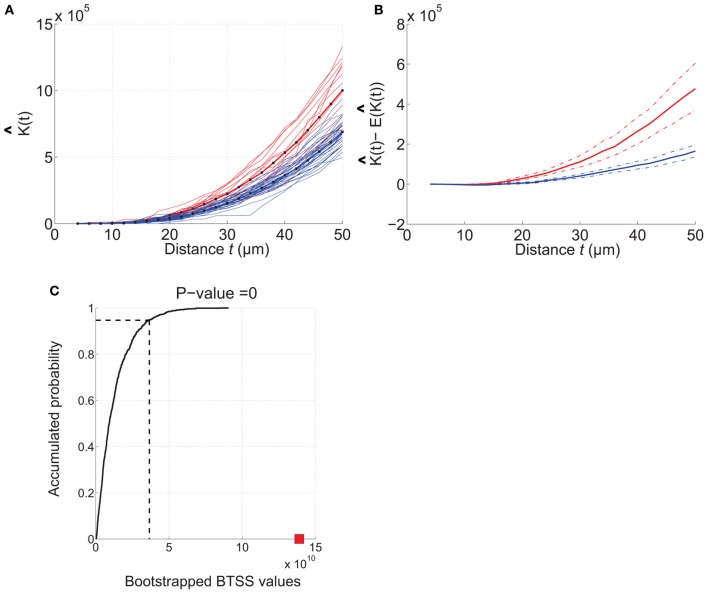
**Example of how RipleyGUI can be used to compare two different cell distributions.** Etv-pyramids in visual cortex (red lines) and etv-pyramids in barrel cortex (blue lines). **(A)** The estimated *K*-function for etv-pyramids in visual (vc-etv) and barrel cortex (bc-etv). **(B)** Average of estimated *K*-function with 95% confidence interval for etv-pyramids in visual and barrel cortex. **(C)** The BTSS value for the experimental data (red square) is larger than the BTSS value at the 0.95 quantile of the accumulated probability distribution. The probability that the compared test distributions are from the same underlying distribution is thus less than 5%. These types of graphs can be generated with the *RipleyGUI*.

The mouse brain samples investigated in the present study were etv-expressing layer 5A pyramidal neurons projecting to striatum [corticostriatal cells; etv-pyramids (Groh et al., 2010)] sampled from the somatosensory barrel cortex and visual cortex. Confocal images were acquired from coronal slices 50–100 μm thick (Figure 3). We chose to analyze for *t*-values up to 50 μm to get estimations for the *K*-function on a varying scale. However, the most stable results for Ripley's *K*-function are for *t*-values smaller than 0.25 times the shortest side of the volume (Ripley, 1988; Diggle, 2003; Costa et al., 2007). The distributions of genetically labeled cells (etv-pyramids) were compared in two different sensory cortices. One aim for such a comparison could be to investigate if local factors influence the structural arrangement, and thus presumably the organization of these cell types in microcircuits. The distribution of etv-pyramids in both somatosensory barrel cortex (Jafari-Mamaghani et al., 2010) and visual cortex (Figures 4, 5) differs significantly from CSR distributions with the same size and intensity. In the somatosensory barrel cortex the sample volume was layer 5A and in the visual cortex it was layer 5 (Groh et al., 2010). The distribution of cells is thus only analyzed with respect to the organization within a specific layer (Figure 3). Figure 4A shows the estimated *K*-function for all the distributions in the experimental data set (blue lines) and all the simulated distributions following CSR (red lines) generated to compare with the experimental data. From visual inspection one can infer that if the different colored lines are separated it is likely that one can discard the hypothesis that the target sample data is based on CSR. The statistical analysis on the existence of any difference between the estimated *K*-functions obtained from the sample data and the distributions following CSR is shown in Figure 4B. This difference is calculated as the fraction of the $\hat{K}$-functions following CSR simulation that are further from *E*[$\hat{K}$(t)] than the sample set's average $\hat{K}$-function. When this is less than 0.05 (the black line), we can discard randomness on a significance level of 0.05. In general the experimental data has negative values for small *t*-values (<15 μm) when estimating $\hat{K}$ (t) – *E*[$\hat{K}$ (t)]. When this difference is negative it indicates inhibition, but for these small *t*-values the “inhibition” is caused by the cell size since no cells can be closer to each other than their diameter. While analyzing the $\hat{K}$-function one must thus consider the diameter of the neurons under investigation.

It is important to keep in mind that the deviations from CSR might be caused by many different factors. If different parts of the measured distribution have different densities, this will result in an aggregated pattern, although it is not caused by actual clusters. Even when stationarity can be guaranteed, we cannot know anything about the underlying process that causes the aggregation. The only certain conclusion is that the sample distribution deviates from CSR. A possible explanation to the aggregated pattern in this data is that it was sampled over column borders. As the cell density is slightly higher in the barrel column than the septa for the etv-pyramids (Groh et al., 2010) the assumption of stationarity is not entirely fulfilled in this area.

#### Comparing two experimental cell distributions

The analysis of each data set [etv-pyramids in barrel cortex (bc) and visual cortex (vc)] thus shows that they are all distributed with a more or less strong tendency to be aggregated (Figures 4, 5). Using *RipleyGUI* one can test if the *K*-functions from two experimental distributions are different using the between-treatments sum of squares, BTSS (see User Manual, and below). In Figure 5A the estimated *K*-function of the etv-pyramids in visual and barrel cortex are plotted. In Figure 5B the average of $\hat{K}$ (t) – *E*[$\hat{K}$ (*t*)] is displayed with a 95% confidence interval. The non-overlapping confidence intervals after *t* = 20 μm mean that 95% of the bc-etv population does not overlap with 95% of the vc-etv population after *t* = 20 μm (and *vice versa*). A more rigorous test, however, of statistical significance between two sample groups can be performed by utilizing the between-group statistics and the BTSS test. In plots of between-group comparisons (Figure 5C), the red square shows the BTSS value for the real sets and the black curve the accumulated probability distribution under the null hypothesis (by bootstrap resampling). The BTSS value for the between-group comparison is calculated over the entire range of *t*-values for the null hypothesis that the two sets stem from the same underlying spatial distribution. This BTSS value (the red square) is beyond the 0.95 quantile of the BTSS distribution based on the BTSS values under the null hypothesis (solid black line). Thus, the probability that the BTSS value based on the actual samples belongs to the bootstrapped distribution is less than 5% and the two sets are significantly different at 5% significance level.

The aim of this analysis was to show how *RipleyGUI* can be used to compare two experimental distributions, the statistical test that can be used and how the results can be interpreted. The results show that the structural organization of a given population of genetically labeled neurons can differ in two sensory cortices. This difference in spatial soma distribution in combination with the differences in neuron morphology (Groh et al., 2010) could indicate that these neuron types are organized according to different structure-function relationship principles in the two different sensory cortices. Larger degrees of aggregation thus means in this case that etv-pyramids in visual cortex are more packed within a sphere with a radius of ~20 μm than expected from a CSR distribution, whereas for etv-pyramids in barrel cortex this is the case only for a bigger sphere with radius ~30 μm. How these changes influence connectivity remains to be investigated combining both experiments and modeling.

## Discussion

We describe a MATLAB-based software for analyzing the spatial distribution of neurons in 3D. The program has a graphical user interface making it easy to use without any MATLAB programing experience. The software is an important addition to a growing arsenal of computer aided programs (http://www.spatstat.org/; Perry, 2004; Wearne et al., 2005; Eglen et al., 2008; Rosenberg and Anderson, 2011) for the analysis of large quantities of structural data that is becoming available (Heintz, 2004; Jones et al., 2009; Berlanga et al., 2011). The use of the method is exemplified by analyzing the distribution of genetically labeled layer 5 corticostriatal cells. We show how this data can be interpreted to indicate differences in the spatial organization of layer 5 pyramidal cells in visual compared to barrel cortex. Conclusive evidence for these differences would, however, require data from large sample regions to overcome possible confounding factors such as non-stationarity and non-uniform sample regions. The developed software tool in combination with experimental techniques that enables physiological measurements from genetically identified neurons (Groh and Krieger, 2011) ensures that structure-function relationships can be examined in great detail.

## Availability and requirements

Operating system(s): Platform independent (tested on Windows XP, and VISTA; Linux Ubuntu; Mac OS X 10.4–10.8). Programing language: MATLAB. Other requirements: MATLAB 7 or higher, Statistics toolbox. License: *RipleyGUI* is distributed free under the conditions that (1) it shall not be incorporated in software that is subsequently sold; (2) the authorship of the software shall be acknowledged in any publication that uses results generated by the software; (3) this notice shall remain in place in each source file.

## Author contributions

Kristin Hansson and Mehrdad Jafari-Mamaghani wrote the modeling code, validated and tested the software. Kristin Hansson designed the program and wrote the user guide. Kristin Hansson analyzed the experimental data. Patrik Krieger conceived the project and refined the software requirements. Kristin Hansson, Mehrdad Jafari-Mamaghani, and Patrik Krieger wrote the paper.

### Conflict of interest statement

The authors declare that the research was conducted in the absence of any commercial or financial relationships that could be construed as a potential conflict of interest.

## Abbreviations

CSR: Complete Spatial Randomness
GUI: Graphical User Interface
BTSS: Between-Treatment Sum of Squares
$\hat{K}$: estimated value of the K-function
*E*[$\hat{K}$(*t*)]: expected K-function
EGFP: enhanced green fluorescent protein.
