# Supplementary material for: RipleyGUI: software for analyzing spatial patterns in 3D cell distributions
Source: Front Neuroinform. 2013 Apr 9;7:5. doi: 10.3389/fninf.2013.00005 (PMC3620507; doi:10.3389/fninf.2013.00005)
Supplement: Supplementary file 1 [file Presentation1.PDF]

RIPLEYGUI USER MANUNAL

KRISTIN HANSSON, MEHRDAD JAFARI-MAMAGHANI, PATRIK KRIEGER

Contents

1. Getting started 2
1.1. Requirements 2
1.2. Installation 2
1.3. Saving the state 2
2. Working with distributions 3
2.1. Suitable distribution properties 3
2.2. Creating distributions 4
2.3. Loading a distribution from file 7
2.4. Ripley's K-function 8
2.5. Comparing with CSR 10
2.6. Station 11
3. Working with sets of distributions 12
3.1. Adding and removing distributions to a set 12
3.2. Set information 13
3.3. Station and Divide 13
3.4. Estimating K-functions 13
3.5. Bootstrap confidence intervals 14
3.6. Between-group comparisons 14
3.7. Compare with CSR 15
3.8. Comparing sets 15
4. Advanced 16
4.1. Exporting to workspace 16
4.2. Running the program from the command window 17
4.3. For Developers 18

1. Getting started

1.1. **Requirements.** RipleyGUI has been developed using MATLAB 7.1. The only requirement to run RipleyGUI is to have MATLAB installed, preferably version 7.0 or later. Statistics toolbox is required.

RipleyGUI has been tested on Windows XP, Windows Vista, Mac OS X and Linux Kubuntu. Nevertheless, given the cross-platform nature of MATLAB, it can be used with any Unix, Macintosh or Windows environment.

1.2. **Installation.** Download the file `RipleyGUI.zip` and unzip it. Add the folder to your MATLAB path.

To run RipleyGUI, type `RipleyGUI` in your MATLAB command window. You should see the window in figure 1.

Figure 1. The opening screen of RipleyGUI

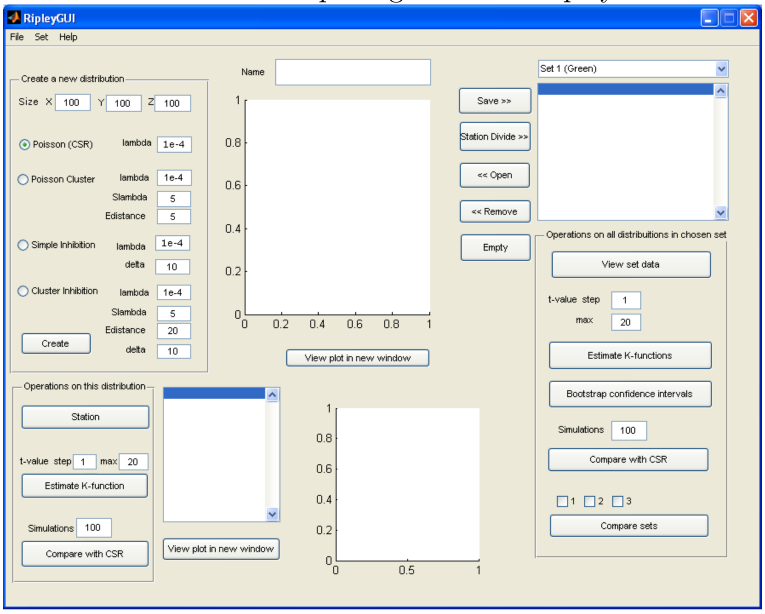

1.3. **Saving the state.** At any time all distributions and calculated properties in RipleyGUI can be saved using `Save GUI State` in the `File` menu or `ctrl-S`. Choosing `Save` the user can choose a new folder and a new file name. Choosing an existing file name will overwrite the old data (see also section 3.1).

To open previously saved work, go to `File` then `Open GUI state` or `ctrl-O`.

2. Working with distributions

The left part of RipleyGUI is for working with single distributions (Figure 2).

Figure 2. Working with single distributions

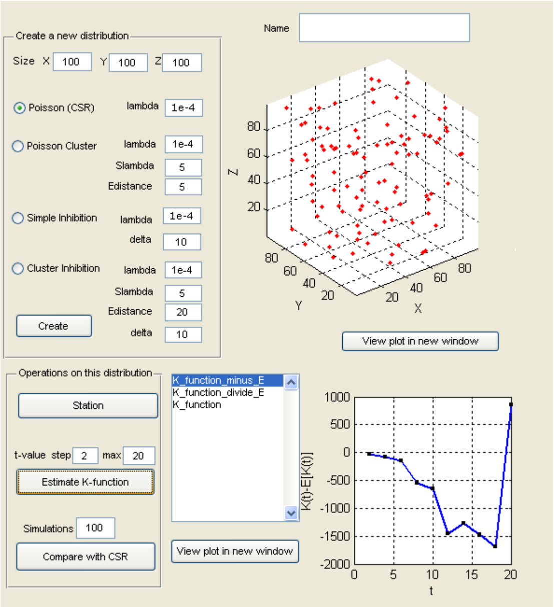

2.1. **Suitable distribution properties.**

The algorithm used to estimate Ripley's K-function assumes that the distribution is *stationary*. That is, the underlying process can be regarded identical in any arbitrary region of the sample domain. The stationarity requirement may not be fulfilled for instance if the intensity is depending on the position in the volume. In figure 3 two examples of non-stationary distributions are shown.

Figure 3. Non-stationary distributions, unsuitable for analysis with RipleyGUI

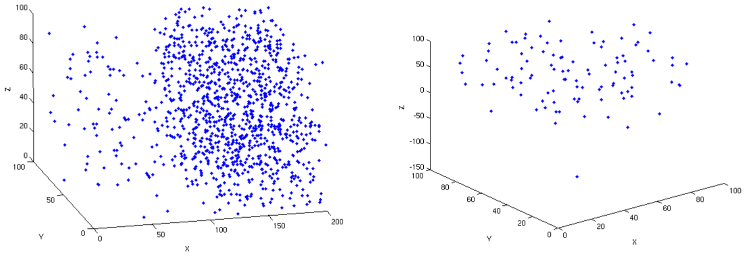

(a) Intensity is non-uniform. Lower intensity in left part of the volume.

(b) a single event expands the boundaries

The distribution region should not be too small, or contain too few events. As a limit, t should not be bigger than 0.25 times the shortest side and the distribution should contain at least 30 events.

2.2. **Creating distributions.** RipleyGUI can create four types of distributions, each with its parameter set. This is useful for exploring different types of spatial point patterns and their K-functions.

To create a new distribution, choose the desired distribution type and its corresponding properties and click `Create`. To rotate or save the distribution, use `View plot in new window`.

- Poisson (CSR).

  In the Poisson Distribution, events are placed randomly and independently in the 3D region. These distributions are representations of complete spatial randomness (CSR). An example is shown in figure 4.

FIGURE 4. A distribution following generated by RipleyGUI Poisson

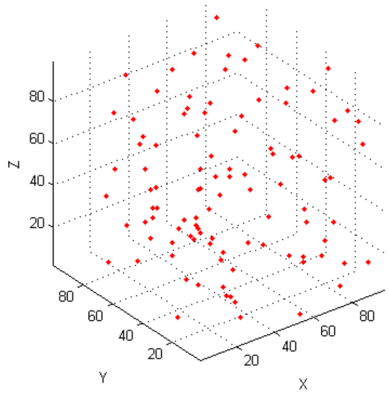

  - `Lambda`

    Lambda is the intensity of the process. The total number of events depends on lambda and the size of the volume. $nr\ of\ events = \lambda \cdot V$. The distribution is conditioned on the intensity.
- Poisson Cluster

  In a clustered, or aggregated, distribution events are closer to their neighbours than expected. A Poisson Cluster distribution is created from randomly distributed parent events, which independently from each other create offspring events.

  Seeding locations of the offspring is independent and identical. Only the offspring are part of the final distribution. Offspring with a position outside the region are placed on the other side of it, that is, they are wrapped.

  An example of a clustered distribution is shown in figure 5.
  - `Lambda`

    Lambda is the intensity of the process. The total number of events depends on lambda and the size of the volume. $nr\ of\ events = \lambda \cdot V$. The distribution is conditioned on the intensity.
  - `Slambda`

FIGURE 5. A clustered distribution generated by RipleyGUI Poisson Cluster

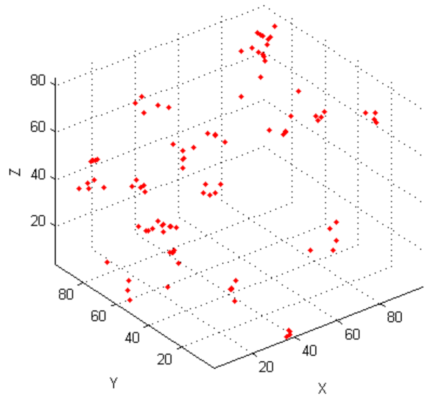

Average number of offspring events per parent event. The number of parent events ρ is given by $\lambda = S \cdot \rho$. For each offspring event a parent event is chosen at random.

– `Edistance`

`Edistance` is the average distance from parent event to offspring events. Each offspring event is placed randomly on the surface area of a sphere with radius E, where E is drawn for each offspring event from a exponential distribution with mean `Edistance`.

- Simple Inhibition

In an inhibited, or sparse, distribution events are less likely to appear close to other events. A simple inhibition distribution is created through generation of independent events where any event closer than a certain distance to an earlier event is discarded. New events are generated until the desired intensity is reached.

Note that the constraint on event proximity limits the maximum possible amount of events. The space reserved by each event is a sphere with radius $\frac{\delta}{2}$, where δ is the minimum distance between events. Volume of the sphere is $V = \left(\frac{\delta}{2}\right)^3 \cdot \frac{4}{3}\pi = \delta^3 \cdot \frac{\pi}{6}$.

Let τ be the packing intensity, the part of the total volume covered by such spheres. The maximum packing intensity differs between distributions, depending on how optimal the random placement turns out. In a worst-case scenario, the distance between events would approach 2δ thus creating an empty space with width approaching δ. RipleyGUI bases the maximum τ allowed on a model where each event occupies a cube with side 2δ. This gives a maximum packing intensity of $\frac{\delta^3\pi}{6}/8\delta^3 = \frac{\pi}{48}$, which is less than the worst-case scenario as the distance between events in the model is more than 2δ in some directions. This margin is intended to speed up the distribution generation, as randomly placing an event in a close-packed distribution might cause time-consuming rejections.

An example of an inhibited distribution is shown in figure 6.

FIGURE 6. An inhibited distribution generated by RipleyGUI Simple Inhibition

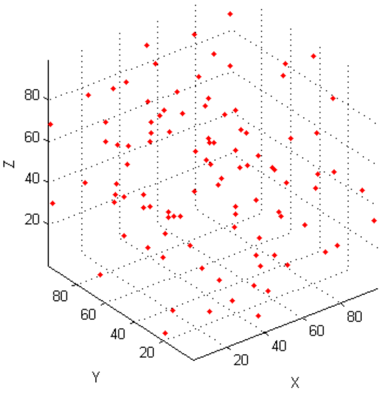

- `Lambda`
  Lambda is the intensity of the process. The total number of events depends on lambda and the size of the volume. $nr\ of\ events = \lambda \cdot V$. The distribution is conditioned on the intensity.
- `Delta`
  Delta is the minimum distance between two events. Any event within this distance is rejected and a new event is generated.

- Inhibited Cluster
  This distribution combines the properties of inhibited and clustered distributions. While events tend towards each other according to the `Edistance` and `Slambda` parameters, they cannot be closer than `delta`.
  An example of an inhibited clustered distribution is shown in figure 7.

FIGURE 7. A clustered and inhibited distribution generated by RipleyGUI Inhibited Cluster

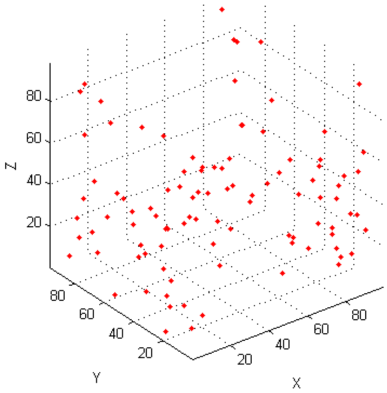

- `Lambda`
  Lambda is the intensity of the process. The total number of events depends on lambda and the size of the volume. $nr\ of\ events = \lambda \cdot V$. The distribution is conditioned on the intensity.
- `Slambda`
  Number of offspring events per parent event. The number of parent events ρ are given by $\lambda = S \cdot \rho$.
- `Edistance`
  `Edistance` is the average distance from parent event to offspring events. Each offspring event is placed randomly on the surface area of a sphere with radius E, where E is drawn from a exponential distribution with average `Edistance`.
- `Delta`
  Delta is the minimum distance between two events. Any event within this distance is rejected and a new event is generated.

2.3. **Loading a distribution from file.** RipleyGUI can read files of ascii format listing event positions. The format of the file should be three columns separated by for instance comma, tab or space. The first, second and third column should contain x, y and z coordinates respectively. Choosing `File` and then `Load` the default setting for files to import is `*.ascii`, but if instead in `Files of types` in the dialog `Pick a file`, `All files` is chosen then both `.txt` and `.csv` files can be imported. In contrast choosing `set->Load ascii folder` then only the ascii files in that folder are imported. The reason for this is that it allows the user to keep in the same folder other files, for example comments which are usually in `.txt` format, and `.csv` files which are often exported from Excel. In these cases where the user wants to import all files in a folder then to import a `.csv` or a `.txt` file one must simply rename them to `.ascii`. Two demo distributions are provided with RipleyGUI in the folder `Demo`.

TABLE 1. Example format of input file

x_1,	y_1,	z_1
x_2,	y_2,	z_2
...	...	...
x_i,	y_i,	z_i
...	...	...
x_n,	y_n,	z_n

To load a distribution, go to the `File` menu and choose `Load ascii distribution` or use `ctrl-L`. Navigate to the file and click `Open`. The distribution is shown with the filename as its name (Figure 8).

2.4. **Ripley's K-function.** Ripley's K-function compares the intensity at a range of localities with the global intensity and hence gives a measure of deviation from the global intensity. It can be expressed as

$$K(t) = \frac{\textit{The expected number of cells within distance t of an arbitrary cell}}{\textit{The total cell intensity}} \quad (1)$$

FIGURE 8. An demo distribution provided with RipleyGUI

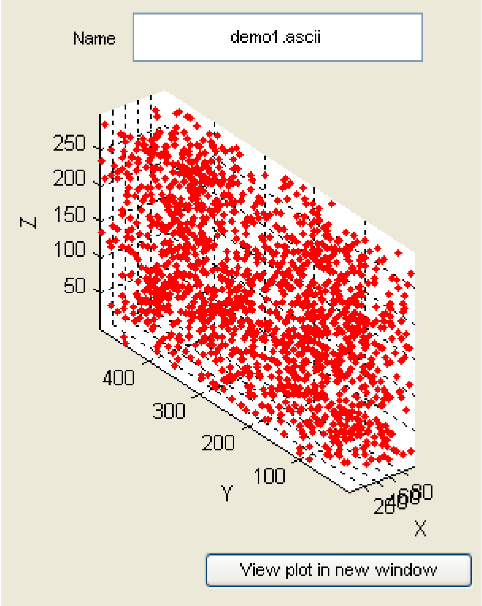

The total cell intensity is estimated as

$$\hat{\lambda} = \frac{n}{V} \tag{2}$$

where n is the number of cells in region V.

The K-function is estimated as

$$\hat{K}(t) = \frac{\hat{E}[N(V_t)]}{\hat{\lambda}} = V\frac{\sum_{i=1}^{n} e_i(t)^{-1} \sum_{j \neq i} I[D(i,j) \leq t]}{n^2} \tag{3}$$

where V is the total volume of the distribution, V_t is a spherical neighbourhood of radius t, n is the number of events, $e_i(t)$ is the edge correction term for event i, I is the indicator function, $D(i,j)$ is the Euclidian distance from event i to j and $N(\cdot)$ is a counting process.

The edge correction term compensates for those parts of the sphere V_t that are outside the sample distribution region and thus cannot include any events.

$$e_i(t) = \frac{Volume\ of\ part\ of\ V_t\ that\ is\ inside\ the\ volume}{Volume of V_t} \tag{4}$$

Preferably, the highest t value should be less than 0.25 times the smallest side.

Assuming a distribution is following CSR and is stationary, the expected value of the estimated K-function is

$$E[\hat{K}(t)] = E\left[\frac{\hat{E}[N(V_t)]}{\hat{\lambda}}\right] = \frac{\lambda V_t}{\lambda} = \frac{4\pi t^3}{3} \tag{5}$$

To estimate Ripley's K-function for the current single distribution, choose desired maximum value and step size for t and click `Estimate K-function` in the field `Operations`

`on this distribution`. A list of available plots and a plot is shown. The available plots and their interpretation are as follows.

- `K function minus E`

  This shows $\hat{K}(t) - E[\hat{K}(t)]$ where $\hat{K}(t)$ is the estimation for the current distribution and $E[\hat{K}(t)]$ is the expected value for a distribution following CSR (Equation 5).

  If the current distribution follows CSR, the values will be around 0, if it is aggregated values will be positive and if it is inhibited values will be negative. Note that since the estimation is based on only one distribution the result may vary. Examples are shown in figure 9.

FIGURE 9. $\hat{K}(t) - E[\hat{K}(t)]$ for some different distributions

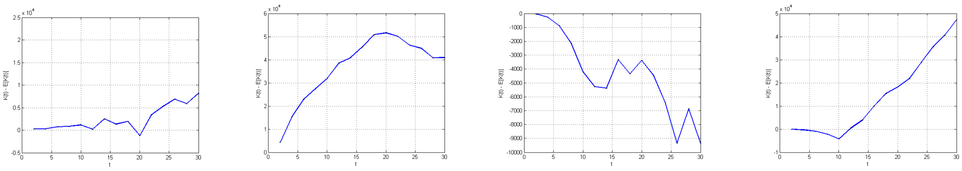

(a) CSR (b) Cluster (c) Inhibition (d) Clustered Inhibition

- `K function divide E`

  This shows $\frac{\hat{K}(t)}{E[\hat{K}(t)]}$ where $\hat{K}(t)$ is the estimation for the current distribution and $E[\hat{K}(t)]$ is the expected value for a distribution following CSR (Equation 5). See figure 10.

  If the current distributions follows CSR, the values will be around 1, if it is aggregated it will be very high but approach 1 for high values of t and if it is inhibited it will be 0 f $t \leq \delta$ and then increase and eventually approach 1. This type of representation makes is especially useful for distinguishing inhibition. To get a feeling for the clustering it is often helpful to inspect $\hat{K}(t) - E[\hat{K}(t)]$ as well as $\frac{\hat{K}(t)}{E[K(t)]}$.

FIGURE 10. $\hat{K}(t)/E[\hat{K}(t)]$ for some different distributions

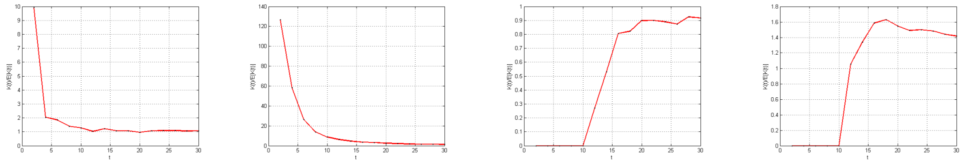

(a) CSR (b) Cluster (c) Inhibition (d) Clustered Inhibition

- `K function`
  This shows only the $\hat{K}$-function and will increase when t increases. For inhibited distributions $\hat{K}(t)$ will be 0 as long as $t \leq \delta$. This plot is more useful when comparing between various distributions.

2.5. **Comparing with CSR.** To quantify the deviations of a distribution from CSR and test to what degree the difference has significance, use the `Compare with CSR` button.

This will generate distributions with the same λ as your distribution and estimate their K-functions. You choose how many distributions to simulate in the `simulation` field. At least 100 distributions are necessary for a reliable result, estimating K-functions for these might be time-consuming.

In the list of available plots two new options will be appear.

FIGURE 11. In (a) one sample distribution (red line) is compared to 100 simulations of CSR (blue lines). For $t < 13$ the estimated K-function (red line) is below the simulated K-functions (blue lines). This graphical indication of spatial point pattern inhibition (section 2.2) can be statistically tested (b) using the p-values obtained from the 95% confidence bounds.

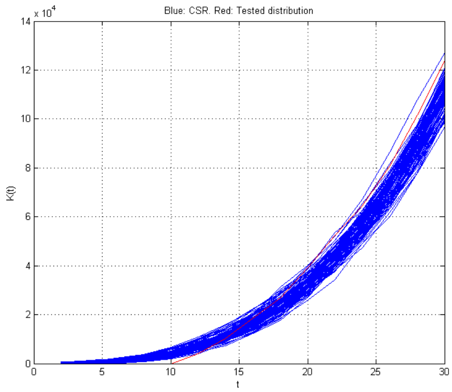

(a) Compare K function

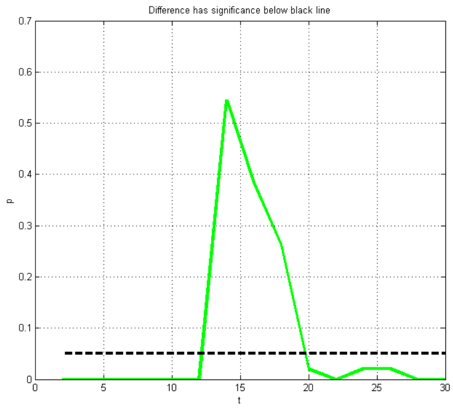

(b) Compare P value

- `Compare K function`
  This shows the estimated $K(t)$ for the generated distributions following CSR as blue lines and the estimated $K(t)$ for the sample distribution as a red line. If the red line lies above the blue lines, this indicates aggregation. If the red lines lies below the blue lines, this indicates inhibition. See figure 12(a).
- `Compare P value`
  This plot shows the significances of the difference between $K(t)$ for each value in the t-vector. The green line shows how likely it is that the current distribution is generated by a completely random distribution. See figure 12(b).

If this is less than 5% it can be rejected with 95% confidence that the current distribution is following CSR. If this indicates a tendency towards aggregation or inhibition is told by the `Compare K function` plot.

2.6. **Station.** The `Station` button will rotate the coordinate system, minimising the volume needed to contain the events in the distribution. A good illustration of how this works is shown in figure 12. Read further about the `Divide` function in section 3.3.

FIGURE 12. Using the Station function on the demo2.ascii distribution.

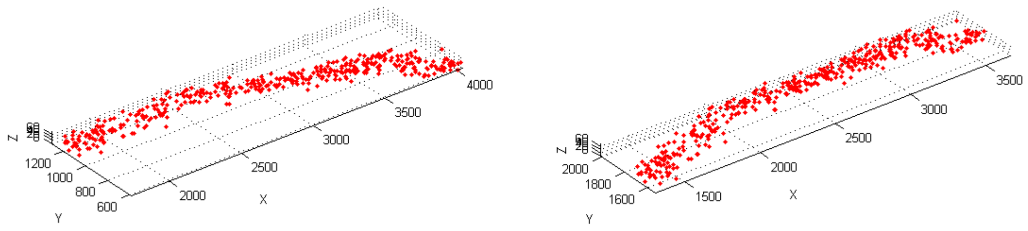

(a) Before (b) After

3. Working with sets of distributions

Working with sets of sample distributions more reliable results will be obtained. The right part of RipleyGUI is designed for this purpose.

RipleyGUI allows working with three sets simultaneously, switch between them with the drop-down menu at top. Default is to work with `Set 1 (Green)`. Each set has a colour associated to it that will be used for plotting all functions related to the set.

Figure 13. Working with sets of distributions

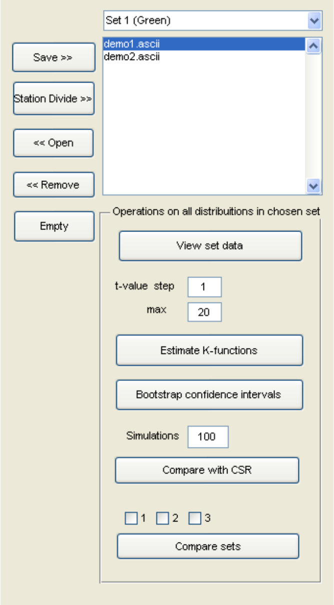

3.1. Adding and removing distributions to a set.

The button `Save` adds the current distribution in the left part of RipleyGUI to the currently selected set. You need to specify a unique name in the `Name` text-box. If using a name already used in the selected set, the old distribution will be replaced.

The button `Open` transfers the selected distribution to the left side of RipleyGUI.

The button `Remove` erases the selected distribution from the set that is currently analyzed. The file is thus only removed for the analysis but the file is not deleted. If the K-function of the set you open is already estimated the values will be shown in the left side K-functions plot. This is a way to explore the K-function of single distributions within a set.

To add more than one distribution at once, use the `Set` menu. `Add 20 to set` will create 20 distributions using the settings from the left side of RipleyGUI and add them all to the currently selected set. `Load ascii Folder` lets you navigate through the file system and pick a directory. All distributions in files ending with .ascii will be added to the set. The distribution files should have the format described in section 2.3.

The button `Empty` will remove all distributions from the currently selected set.

3.2. Set information. The button `View set data` shows some information about the current set; number of distributions, average intensity, size and whether all K-functions have been estimated.

3.3. Station and Divide. For datasets containing non-uniform and/or elongated sample domains, the `Divide` function can be used to obtain more uniformly shaped sample domains (Figure 14). While the `Station` function (section 2.6) rotates a spatial point pattern distribution on the *2D* plane to better fulfill the assumption of stationarity, the `Divide` function creates subdomains of data along the longest dimension of the original sample domain. If the longest and second-longest sides of the domain are equal, divide will have no effect.

FIGURE 14. Using divide twice on the demo1.ascii distribution

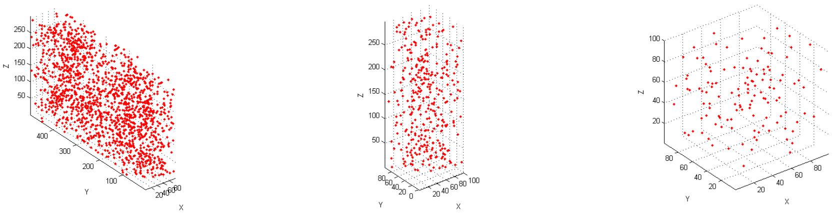

(a) Before (b) First Divide (one of the pieces) (c) Second Divide (One of the pieces)

3.4. Estimating K-functions. When clicking the button `Estimate K-functions` the K-functions for all distributions in the set will be estimated. A new window will display plots for $\hat{K}(t)$, $\hat{K}(t) - E[\hat{K}(t)]$ and $\hat{K}(t)/E[\hat{K}(t)]$. The weighted average of the distributions will be drawn with a thicker line in each plot. See figure 15 for an example.

FIGURE 15. Estimated K-functions for all plots in the current set. Weighted average of the estimated K-functions is shown in a thicker line.

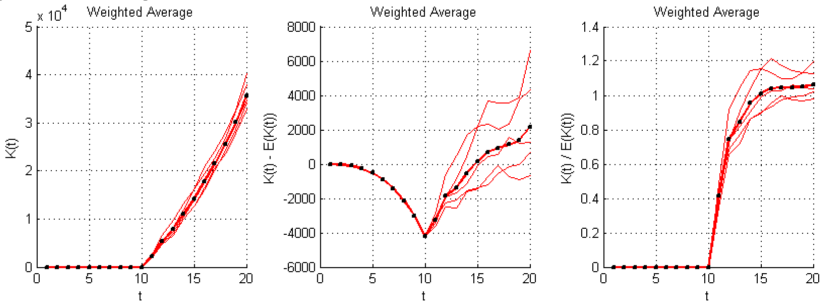

The K-functions are saved when estimated. If for instance a new distribution is added to a set for which K-functions already were estimated, only the K-functions for the new distribution will have to be re-estimated. The estimated $\hat{K}$-functions are saved only for the latest used t-vector. If `t-step` or `t-max` is changed all the K-functions will have to

be re-estimated (the only exception is if `t-step` remains the same and `t-max` is changed to a smaller value).

The average of the estimated K-function is weighted so that distributions with more events influence the outcome proportionally.

$$K_{average}(t) = \frac{\sum_{i=1}^{nrofdistributions} K_i(t) * n_i}{\sum_{i=1}^{nrofdistributions} n_i} \quad (6)$$

where n_i is the number of events in the distribution.

The weighted average will show a more representative behaviour than the K-function of a single distribution. Read more in section 2.4 section about interpretation of the shape of the curve.

3.5. **Bootstrap confidence intervals.** When a set contains a large number of distributions the $\hat{K}$-functions displayed may overlap and visually interpreting the plot becomes more difficult. The confidence intervals are a more convenient way of visualising the amount of variation from the average $\hat{K}$-function. They show the interval within which 95 % of the realisations from the distribution is expected to fall. Use the button `Bootstrap confidence intervals` to show these intervals for $\hat{K}(t)$, $\hat{K}(t) - E(\hat{K}(t))$ and $\hat{K}(t)/E(\hat{K}(t))$. See figure 16 for an example.

Figure 16. Bootstrapped confidence intervals and the weighted average.

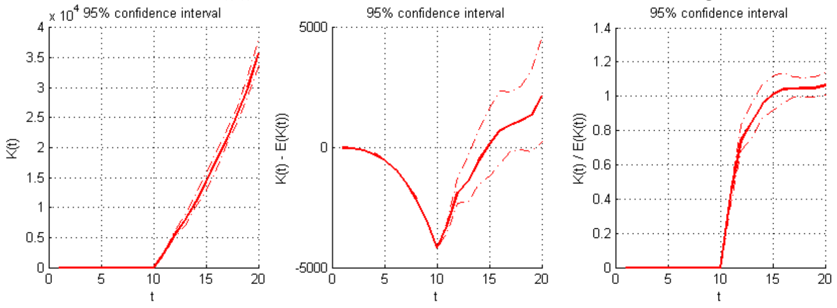

3.6. **Between-group comparisons.** Between-group comparisons are used in `Compare with CSR` and `Compare sets`. They are based on the hypothesis that two sets are based on identical underlying point pattern distributions. Under this hypothesis, replacing a distribution in a set with a distribution from the other set should not affect the weighted average $\hat{K}$-functions.

To verify this hypothesis, sets with the same number of samples as the original set, chosen randomly from both sets are created using replacement. This procedure is done 5000 times. A score using a modification of sum of squares' function (BTSS), is calculated for each of the 5000 resamplings and the real sets. The verification of the hypothesis is then reduced to investigating whether or not the score based on the real sets is likely to have been produced by the scores under the hypothesis.

In plots of between-group comparisons, the red square shows the BTSS value for the real sets and the black curve the accumulated probability distribution of the resamplings.

If the p-value is less than 0.05, the hypothesis that the compared distribution are from the same underlying point pattern can be rejected with a confidence level of 0.95.

3.7. **Compare with CSR.** The `Compare with CSR` button opens a new window with four plots. The weighted average $\hat{K}$-function from `Estimate K-functions` is compared with the $\hat{K}$-functions for a set following CSR. The set following CSR is generated with the average size and intensity of the distributions in the set.

A plot like the one in figure 17 will be shown. The upper left plot shows the estimated K-functions and their weighted average from the current set together with the estimated K-functions following CSR.

The upper right plot shows the P-values, an indication of whether the current set is follows the CSR pattern. When below 0.05 it can be rejected with 95% confidence that the set follows CSR.

In the lower left plot the weighted average from the set distributions and the CSR distributions are shown with their bootstrapped confidence intervals. Where the intervals are completely separated it is an indication that the distributions are different. This information can be confirmed by the p-value in the BTSS comparison shown in the lower right plot (see section 3.6).

FIGURE 17. An set of inhibited cluster distributions compared with 100 simulated CSR distributions

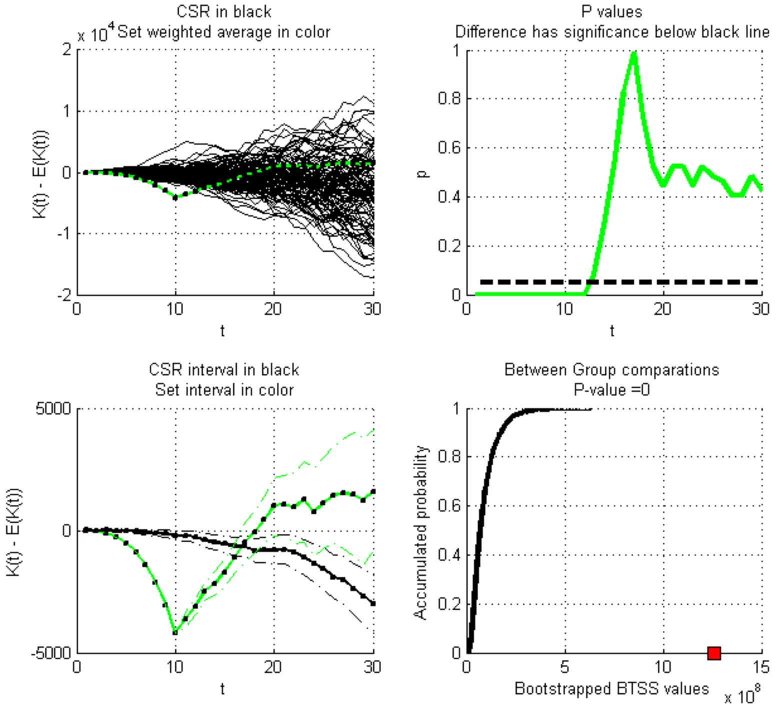

As in the case with a single distribution it is necessary to generate at least a hundred simulations to obtain a reliable result. RipleyGUI saves the set of distributions

following CSR with K-functions. Unless t-values or number of simulations are changed, RipleyGUI will give the option to use the same values for CSR distributions as in previous calculations. This option is suitable when λ and volume are still similar to the current distribution.

3.8. **Comparing sets.** The estimated K-functions and the bootstrapped confidence intervals can be compared between up to three different sets. When the estimation and bootstrapping for more than one set is finished, check the boxes for the relevant sets and click `Compare sets` A plot like figure 18 will be displayed.

The estimated K-function for each set will be displayed in the colour that is assigned to it. Inspecting the graphs will give initial information on whether two sets are the result of different distributions. If the bootstrapped confidence intervals are completely separated, it is an indication of the sets being different, although not a test for significance. Use the BTSS plots (section 3.6) to confirm the significance of the difference between sets.

FIGURE 18. A set of clustered-inhibited distributions (green) and two sets of distributions following CSR (red, blue) compared to each other.

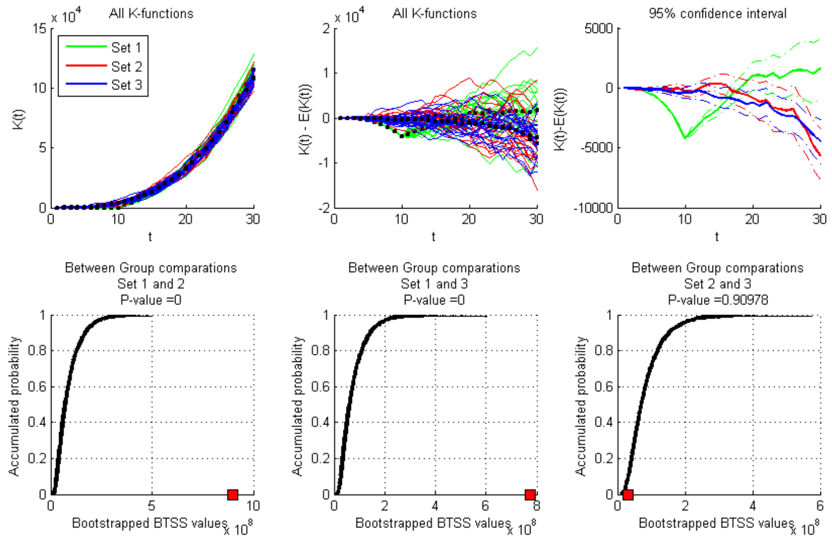

4. Advanced

4.1. Exporting to workspace. To do further calculation not provided within the scope of RipleyGUI you can export the internal data to your MATLAB workspace.

`File -> Export to workspace` will export the single distribution and its estimated K-function. You will reach the data through the variables `ripGUI_dist`, `ripGUI_ripK`, `ripGUI_EK` and `ripGUI_t`. Type their name in MATLAB command window to see how they look. If the K-function is not yet estimated, only the distribution will be exported.

`Set ->Export to workspace` will export the currently available set to workspace. All the information will be in the variable `ripGUI_set1` for set 1 and so on.

`ripGUI_set1` is a cell array where each cell contains a distribution. Use `ripGUI_set1{i}` to reach the i:th distribution. An example of this is shown in figure 19, accessing a distribution will show a list of variables available for the distribution. The variables are reached e.g. with `ripGUI_set1{i}.ripK`.

Figure 19. Some commands looking at exported variables from RipleyGUI

```
Command Window

>> ripGUI_set2{1}

ans =

        X: [133x1 double]
        Y: [133x1 double]
        Z: [133x1 double]
     name: 'glt1515-2.ascii'
     ripK: [1x20 double]
       EK: [1x20 double]
    stepT: 1
     maxT: 20

>> ripGUI_set2{1}.ripK

ans =

  1.0e+004 *

  Columns 1 through 8

         0         0         0         0         0         0         0    0.1446

  Columns 9 through 16
```

4.2. Running the program from the command window. The following functions can also be used from the command window or from a script. Please refer to the `help` function for further information on their use.

- ripleyK_comuse
- create_po_distribution
- create_po_cl_distribution
- create_inhib_distribution
- station
- divide
- pval

4.3. **For Developers.** RipleyGUI has been developed using the `guide` tool in MATLAB. To open the layout, type `guide` in your MATLAB command window and open `RipleyGUI.fig`.

Variables are shared between functions in the `handles` structure.

Future improvements may include the possibility to decide step size and starting point in the t array independently of each other. An interesting new feature would be the possibility to analyse the cross-relationship between different populations in the same volume.
